# RapidArc Dynamic (RAD) multi‐mechanical axis optimization achieves enhanced OAR sparing in cervical cancer: A dosimetric comparison study

**DOI:** 10.1002/acm2.70506

**Published:** 2026-02-17

**Authors:** Xiangyin Meng, Zhiqun Wang, Yiming Zhang, Xingliu Wang, Xiaoshen Wang, Weihua Zhu, Fuqiang Chen, Zhen Zhang, Fengchao Xu, Zhufeng Wu, Bo Yang, Jie Qiu

**Affiliations:** ^1^ Department of Radiation Oncology Peking Union Medical College Hospital Beijing China; ^2^ UMass Chan Medical School Worcester Massachusetts USA; ^3^ Varian Medical System A Siemens Healthineers Company Beijing China

**Keywords:** cervical cancer, OAR sparing, RapidArc Dynamic (RAD), volumetric modulated arc therapy (VMAT)

## Abstract

**Purpose:**

To overcome limitations of conventional VMAT for cervical cancer, specifically restricted OAR sparing due to static collimators and delivery constraints inherent to dynamic modulation, we systematically evaluate RapidArc Dynamic (RAD) technology. This first comprehensive assessment focuses on RAD's multi‐axis coordination for optimized pelvic radiotherapy planning.

**Methods:**

Twenty cervical cancer patients treated with TrueBeam whose energy was 6MV X‐ray were retrospectively analyzed. The prescription was 45 Gy/25Fractions. Seven RAD‐based planning strategies with varying collimator rotation modes (Optimize [OPT] vs. Optimize Between Static Angles [OBSA] vs. Static [STAT]) and aperture sizes (15 vs. 30 cm) and different static angle ports were developed using identical optimization objectives. Re‐optimized clinical plan using Eclipse v18.1. All RAD and clinical plans were normalized so that the target received a certain dose with 95% target coverage for final dosimetric comparison. Dosimetric comparisons employed ANOVA (with LSD post hoc) for parametric data or Kruskal–Wallis's test (with Mann–Whitney *U* post hoc) for non‐parametric data, with significance set at *p* < 0.05.

**Results:**

RAD plans showed slightly inferior conformity and homogeneity compared to clinical plans, they achieved superior dose fall‐off gradients. RAD's advantage was particularly evident in sparing organs at risk (OARs). Specifically, for the bladder, significant reductions were noted in $D_{mean}$(8.29%–13.05%), $V_{20\;\mathrm{Gy}}$(4.04%–26.71%), and $V_{30\;\mathrm{Gy}}$(26.98%–32.46%) compared to the clinical plans. Similarly, for the rectum, reductions in $D_{mean}$(9.21%–15.58%), $V_{20\;\mathrm{Gy}}$(5.03%–14.77%), $V_{30\;\mathrm{Gy}}$(18.24%–28.11%), and $V_{40\;\mathrm{Gy}}$(23.73%–31.22%) were observed. Other OARs also benefited from improved dosimetric parameters. While both the 2ARC+OBSA+15 and 2ARC+STAT+15 plans outperformed other RAD configurations, the 2ARC+OBSA+15 plan generally provided superior results. Notably, although RAD plans overall showed significantly higher small intestine *D_2cc_*, the 2ARC+OBSA+15 configuration achieved comparable doses to clinical plans. In terms of monitor units (MU) and plan complexity, RAD plans were more complex but maintained a high verification pass rate of over 97%.

**Conclusion:**

The preliminary exploration of RAD plans reveals superior capability in sparing the doses of OARs. Moreover, in the RAD plans, the optimization of collimator angles and aperture at 15 cm yields better dosimetric outcomes. Therefore, further exploration of different mechanical axis combinations is of great significance in future studies.

## 1 INTRODUCTION

Cervical cancer is a major cancer that endangers women worldwide. Various treatment modalities are available for cervical cancer, with radiation therapy being one of the key treatments, as approximately 60% of patients receive radiation therapy.^1^ In earlier practices, three‐dimensional conformal radiation therapy (3D‐CRT) was the primary treatment for cervical cancer; however, for locally advanced tumors, it failed to significantly reduce the radiation dose of the bowel or rectum.^2^ Then, intensity‐modulated radiation therapy (IMRT) has become increasingly prevalent in the radiotherapy of cervical cancer, offering superior protection for normal tissues.^3^ Subsequently, volumetric modulated arc therapy (VMAT) emerged, which can achieve similar homogeneity and conformity as IMRT while significantly reducing monitor units (MU) and treatment time,^4^ thus improving treatment efficiency.^5^ Currently, both IMRT and VMAT are the mainstream techniques used in the clinic.

Although both IMRT and VMAT ensure high prescription dose coverage to the target volume and effectively spare OARs, further exploration of hybrid planning approaches is warranted. Hybrid planning balances target dose, treatment time, and MU while better controlling the dose to OARs.^6^, ^7^, ^8^, ^9^ However, hybrid planning requires the design of two separate radiation treatment plans, which may result in variations in execution, such as the distribution of treatment fractions between IMRT and VMAT,^7^, ^9^ or the execution of both plans in a single treatment session.^6^, ^8^ Furthermore, in the context of breast cancer hybrid plans, usually, a specific proportion of the prescribed dose is allotted to the 3D ‐ CRT plan (approximately 60%–80%), with the remaining portion assigned to the IMRT/VMAT plan (roughly 40%–20%). This is referred to as the dose separation ratio. However, a multitude of factors can impact the prescription dose proportion within hybrid plans, including the size and the shape of the target volume, the heart dose, the ipsilateral lung dose, and so forth.^10^ Compared with hybrid plans, optimizing the collimator angle in VMAT is another promising method to improve plan quality. In current VMAT plans, the collimator angle is typically fixed; however, optimizing this angle has the potential to significantly improve plan quality. Depending on the disease type or tumor morphology, choosing the optimal collimator angle can improve RT plan quality,^11^, ^12^, ^13^ and research on emulating dynamic collimator adjustments also yield favorable results.^14^, ^15^

RapidArc Dynamic (RAD), a novel planning and treatment solution, is a feature implemented in the Eclipse Treatment Planning System (Version 18.1, Varian Medical Systems). RAD enables modulation through static‐gantry modulated ports and enables dynamic collimator angle optimization for VMAT technique.^16^ These enhancements expand mechanical axis optimization beyond multi‐leaf collimator (MLC), dose rate adjustments and gantry speed, improving mechanical utilization efficiency.

To date, this is the first study to apply RAD functionality in cervical cancer cases. Using Eclipse v18.1, seven different RAD plans were designed to explore suitable RAD treatment modalities for cervical cancer. These plans were compared with the clinical two‐arc VMAT plan to assess the characteristics of RAD planning.

## 2 MATERIALS AND METHODS

### 2.1 Patient selection

A retrospective analysis was conducted on 20 cervical cancer patients treated with 2ARC‐VMAT using TrueBeam STx (Varian Medical Systems) between April 2023 and October 2024. All patients were randomly selected and anonymized. The median age was 49 years, with a range from 29 to 62 years. Target volumes and OARs were delineated according to ICRU‐83^17^ and the report of RTOG 0418.^18^ The target volumes included the Clinical Target Volume (CTV) and the Planning Target Volume (PTV), with the PTV obtained by extending CTV by 6 mm in the anterior‐posterior and left‐right directions, and by 8 mm in the superior‐inferior direction.^19^ OARs included the bladder, rectum, small intestine, spinal cord and femur head.

### 2.2 Treatment planning

A series of seven RAD plans were generated using Eclipse v18.1 and compared with the clinical plan (RapidArc_2Arcs), which was originally designed in Eclipse v15.6 but re‐calculated and re‐optimized in v18.1 for consistent comparison. All plans were designed by the same medical physicist and optimized for delivery on a TrueBeam linear accelerator. These plans were compared to evaluate differences in dosimetric parameters for both the target volume and OARs, as well as QA pass rates, MU and plan complexity. For the seven RAD plans, three distinct optimization strategies for the collimator angle were implemented: ① Optimize (OPT): The collimator angle was fully optimized within the angular ranges defined by both the arc and static fields, with rotation occurring during the arc field and the collimator remaining stationary during the static field. ② Optimize Between Static Angles (OBSA): This approach defines the start and end angles of collimator for the arc field and fixed collimator angles for the static fields. Optimization of the collimator angle was performed between the start and end of the arc field. ③ Static (STAT): The collimator angle remained fixed during the entire gantry rotation. Additionally, RAD combines VMAT arcs with static angle modulated ports (STAMPs) deliveries. Within the optimization interface, the relative weighting between arcs and ports can be selected across a spectrum from −2 (Arc‐Dominant) to +2 (Static‐Dominant), with intermediate values: −1 (Arc), 0 (Balanced), and +1 (Static). All RAD plans in this study employed the balanced weighting approach (0).Through the application of these collimator strategies, gantry ports, and aperture settings, seven distinct RAD plans were created and compared with the conventional clinical plan. The naming convention for six of the RAD plan groups is as follows: Taking 2 ARC+OPT+30 as an example, 2 Arc indicates the use of two arcs, OPT denotes the optimized collimator angle strategy, and 30 represents a maximum field opening (aperture) of 30 cm in the X‐direction. All five 2 Arc RAD plans utilized 1 STAMP per arc at the starting angel of the arc, which is not explicitly stated in the naming. In contrast, the 1 Arc RAD plan employed 7 ports, hence this detail is included in its naming. Furthermore, another RAD plan is named “RAD_QSG,” which has plan parameters set according to the Varian RAD quick start guide document from https://www.myvarian.com/. The clinical VMAT plan (utilizing two full arcs) for pelvic radiotherapy served as the control plan in this study. Details for each plan configuration are provided in Table 1.

**TABLE 1:** Mechanical modulation parameters of seven RAD plans compared to clinical plan.

Treatment plan	Arc beam angle	STAMPs	Collimator	Aperture (X‐axis maximum field opening)
1 Arc+OPT+7 Ports	181°–179°	0°, 51°, 102°, 154°, 208°, 258°, 309°	Automatic optimization	Automatic (by arcgeometry tool)
2 Arc+OPT+30	181°–179° & 179°–181°	181° for arc1 & 179° for arc 2	Automatic optimization	30 cm
2 Arc+OBSA+15	181°–179° & 179°–181°	181° for arc1 & 179° for arc2	348°–12°	15 cm
2 Arc+OBSA+30	181°–179° & 179°–181°	181° for arc1 & 179° for arc2	348°–12°	30 cm
2 Arc+STAT+15	181°–179° & 179°–181°	181° for arc1 & 179° for arc2	315° & 45°	15 cm
2 Arc+STAT+30	181°–179° & 179°–181°	181° for arc1 & 179° for arc2	315° & 45°	30 cm
RAD_QSG	181°–179° & 179°–181°	245°, 45° for arc1 & 110°, 320° for arc2	Automatic optimization	15 cm
Clinical	181°–179° & 179°–181°	N/A	315° & 45°	Automatic (by arcgeometry tool)

Abbreviations: OBSA, optimize between static angles; OPT, optimize; STAMPs, static angle modulated ports; STAT, static.

All treatment plans were planned with 6MV‐FF photon and Millennium MLC, with dose calculation performed using the Acuros XB algorithm. Optimization was carried out with the Photon Optimizer (PO) algorithm, which incorporates a Direct Aperture (DA) optimization method. This method is combined with an eight‐level Multiresolution (MR) approach, allowing for precise adjustments to control point attributes, such as leaf positions, collimator settings, and dose weightings across different resolution levels.^16^

The PTV prescription was 45 Gy in 25 fractions, and the dose distribution required that at least 95% of the target volume receive 45 Gy, with no significant regions exceeding 107% of the prescribed dose. All plans used the same dose constraints for OARs, including critical structures such as the bladder, rectum, and small intestine. And finally all plans were normalized to PTV to which received 95% prescription dose coverage.

### 2.3 Plan evaluation

Dosimetric evaluation of the target volume was performed using the following metrics: $V_{100\%}$ and $V_{107\%}$. Additional evaluation criteria included the Conformity Index (CI),^17^ Homogeneity Index (HI),^17^ and Gradient Index (GI),^20^ defined by the following Equations (1)–(3).

(1) $$CI=\frac{V_{ref}}{V_{T}}$$

(2) $$HI=\frac{D_{2\%}-D_{98\%}}{D_{50\%}}\;$$

(3) $$GI=\frac{V_{50\%}}{V_{T}}\;$$

where $V_{T}$ is the volume of the target, and $V_{ref}$ is the volume encompassed by the 100% isodose line. $D_{2\%}$, $D_{50\%}$, and $D_{98\%}$ represent the dose received by 2%, 50%, and 98% of the target volume, respectively. $V_{50\%}$ is the volume of the target receiving 50% of the prescribed dose.

For OARs, the evaluated dosimetric metrics included the following: for the bladder, rectum, and bone marrow, they were $D_{mean}$, $V_{20\;\mathrm{Gy}}$, $V_{30\;\mathrm{Gy}}$, and $V_{40\;\mathrm{Gy}}$; for the small intestine, they were $D_{mean}$, $V_{20\;\mathrm{Gy}}$, $V_{30\;\mathrm{Gy}}$, $V_{40\;\mathrm{Gy}}$, and $D_{2cc}$; for the left and right femoral heads, it was $D_{5\%}$; and for the spinal cord, it was $D_{0.1cc}$.

Additionally, non‐dosimetric parameters were evaluated, including the MU, plan complexity, and QA pass rate. For plan complexity, Masi et al. introduced the concept of Modulation Complexity Score (*MCS*)^21^ to quantify the complexity of VMAT plans (*MCS_v_*).^22^ The calculation of the modulation complexity score *MCS_v_* is defined in Equation (4):

(4) $$\begin{array}{cc} MCS_{v} & =\sum_{i=1}^{I-1}\left[\left(\frac{\left(AVV_{cpi}\pm AAV_{cpi+1}\right)}{2}\right.\right.\\ & \left.\left.\times \frac{\left(LSV_{cpi}\pm LSV_{cpi+1}\right)}{2}\times \frac{MU_{cpi,i+1}}{MU_{arc}}\right)\right]\; \end{array}$$

where i is the current control point number. The LSV parameter is defined for each control point considering in each bank the differences in position between adjacent MLC leaves. $LSV_{cpi}$ is the leaf sequence variation coefficient at the current control point i; The $LSV_{cpi}$ is defined in Equation (5) and (6):

(5) $$Pos_{max}\;\mathrm{cp}={\langle max\left(pos_{n\in N}\right)-min\left(pos_{n\in N}\right)\rangle }_{leafbank\;}\;$$

(6) $$\begin{array}{cc} LSVcp= & {\left(\frac{\sum_{n=1}^{N-1}\left(pos_{max}-\left|\left(pos_{n}-pos_{n+1}\right)\right|\right)}{\left(N-1\right)\times pos_{max}}\right)}_{leftbank\;}\\ & \times {\left(\frac{\sum_{n=1}^{N-1}\left(pos_{max}-\left|\left(pos_{n}-pos_{n+1}\right)\right|\right)}{\left(N-1\right)\times pos_{max}}\right)}_{rightbank\;} \end{array}\;$$

where *N* is the number of moving leaves inside the jaws and *pos* is the coordinate of leaf position.

The AAV is calculated as the area defined by apertures of opposing leaves in the single control point normalized to the maximum area in the arc, defined by the maximum apertures for each leaf pair over all CPs in the arc. $AVV_{cpi}$ is the aperture area variation coefficient at the current control point i; The $AVV_{cp}$ is defined in Equation (7):

(7) $$\begin{array}{cc} AAV_{cp}= & \frac{\sum_{a=1}^{A}\left({\left\langle pos_{a}\right\rangle }_{leftbank}-{\left\langle pos_{a}\right\rangle }_{rightbank}\right)}{\sum_{a=1}^{A}\left({\left\langle max\left(pos_{a}\right)\right\rangle }_{leftbank\in arc}-{\left\langle max\left(pos_{a}\right)\right\rangle }_{rightbank\in arc}\right)} \end{array}\;$$

where *A* is the number of leaves in the arc. $MU_{cpi,i\pm 1}$ represents the monitor units for adjacent control points; and $MU_{arc}$ is the MU for the full arc. As depicted by the formulas above, LSV represents the disparity in the MLC leaf sequence. The smaller the difference between adjacent leaves and the smaller the maximum difference of single‐side MLC leaves, the greater the LSV value. A larger LSV value implies a lower complexity for this control point. AAV describes the area change created by the MLC leaves. The smaller the aperture formed by all the leaves on both sides and the larger the area formed by paired leaves, the greater the AAV value. A larger AAV value indicates a lower complexity for this control point. So, a lower $MCS_{v}$ value indicates higher complexity in the plan.

Another factor used to describe plan complexity is the Average Leaf Pair Opening (ALPO).^23^, ^24^ The calculation of $ALPO_{MU}$ is defined in Equation (8):

(8) $$ALPO_{MU}=\sum_{k=1}^{k=K}\frac{\sum_{i=1}^{i=I-1}\left[\sum_{j=1}^{j=J}{\left(\left|X_{R}-X_{L}\right|\right)}_{i,j}\right]\cdot MU_{i,i\pm 1}}{\left(\sum_{i=1}^{i=I-1}MU_{i,i\pm 1}\right)\cdot \sum_{j=1}^{j=J}j}/K\;$$

where K is the total number of treatment fields; I is the total number of control points in each treatment field; J is the number of valid leaf pairs at each control point (defined as leaf pairs with a separation greater than 0.5 mm); and $MU_{i,i\pm 1}$ is the monitor units from one control point to the next. A lower ALPO value indicates higher complexity in the plan.

The QA pass rate was determined by using ArcherQA for plan verification, since our TrueBeam version was 2.7 which cannot support the RAD plan delivery. ArcherQA is a quality assurance tool for radiotherapy plans, which utilizes the Monte Carlo (MC) method for secondary dose calculation, ensuring the safety of the beam delivery. A gamma analysis with criteria of 3% dose difference and 3 mm distance‐to‐agreement was used for the verification.

### 2.4 Statistical analysis

Data analysis was performed using an in‐house statistical analysis script. For datasets that followed a normal distribution with homogeneity of variance, one‐way analysis of variance (ANOVA) was used, with post hoc analysis performed using the least‐significant‐difference (LSD) method. A *p*‐value of less than 0.05 was considered statistically significant, and the data were expressed as $\bar{x}\pm s$. The Kruskal–Wallis test for multiple independent samples was employed where datasets did not meet the assumptions of normality and homogeneity of variance. A *p*‐value of less than 0.05 indicated significant differences, and post hoc analysis was conducted using the Mann–Whitney *U* test for pairwise comparisons. Data were presented as Med(Q1,Q3).

## 3 RESULTS

### 3.1 Patient example

**FIGURE 1:**
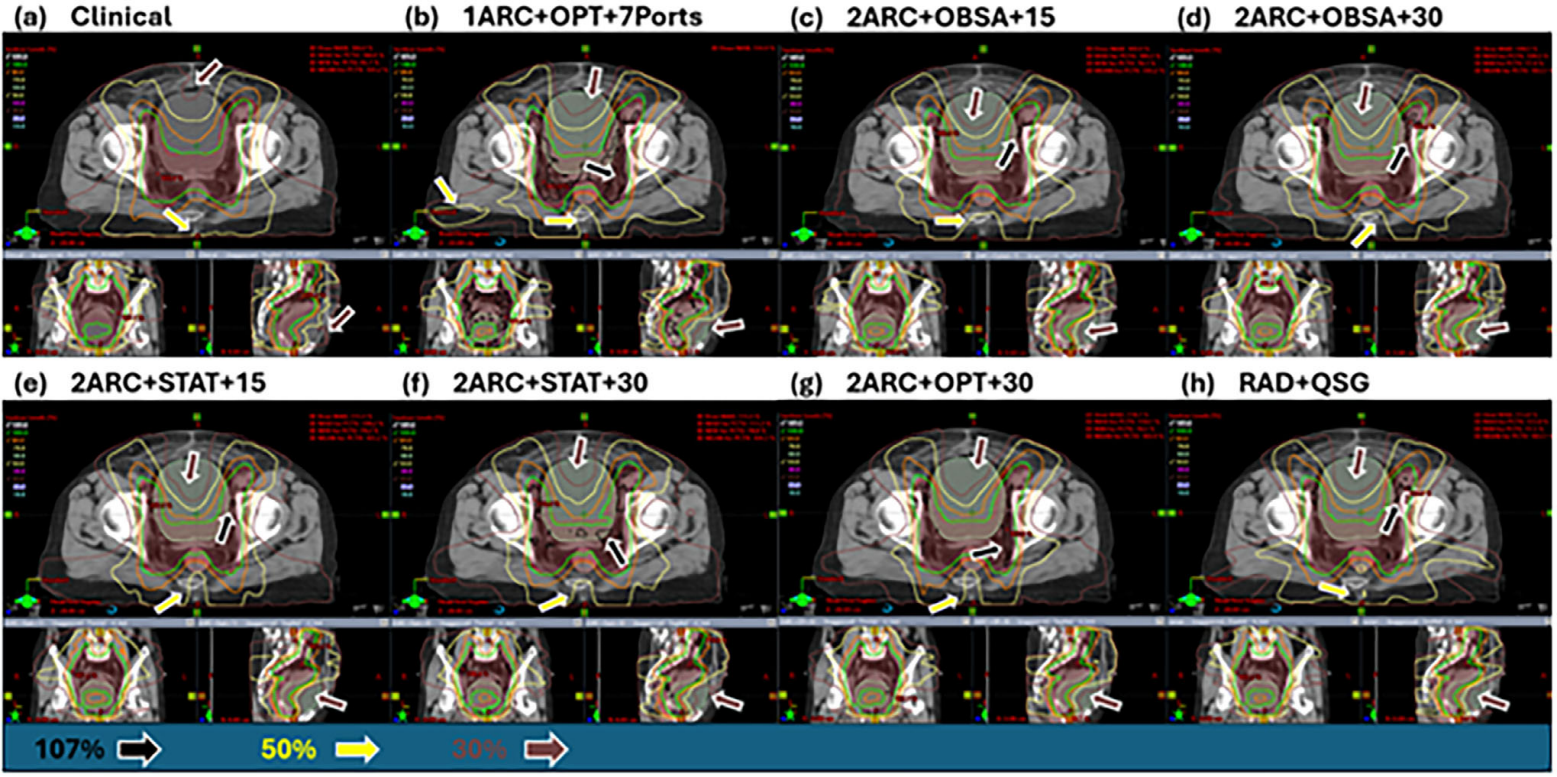
Dose distribution comparison for a specific patient with eight plan variations. As observed, the clinical plan demonstrates an advantage in terms of high‐dose region control, while the RAD plans show superior protection for OARs. Additionally, as the arrows show, the 2ARC+OBSA+15 plan provides effective high‐dose control and the best OAR dose sparing at the same time. Specifically, the red truncated area is the PTV.

**FIGURE 2:**
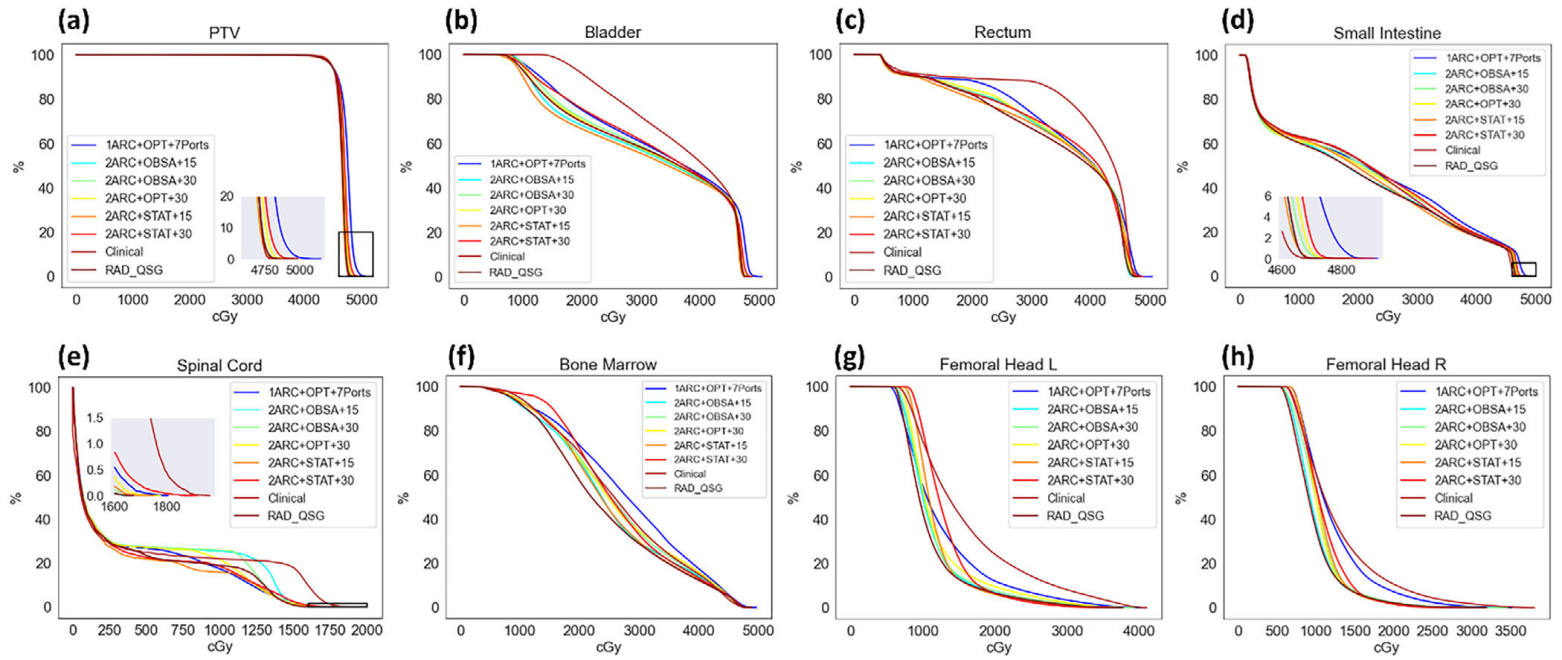
DVH comparison of target volume and OARs for eight treatment plans in a specific patient 6. As shown in (a) and (d), the RAD plans do not show an advantage in PTV and small intestine high dose region. However, within the RAD plans, the 2ARC+OBSA+15, 2ARC+STAT+15, and RAD_QSC plans are closer to the clinical plan. In (e), regarding the maximum dose control of the spinal cord, the RAD plans demonstrate a clear advantage. In (b), (c), (f), (g), and (h) RAD plans exhibit superior OAR dose reduction, with the 2ARC+OBSA+15, RAD_QSC, and 2ARC+STAT+15 plans showing the most prominent benefits.

Figures 1 and 2 illustrate the dose distribution and DVH differences for patient 6. These figures show that RAD plans generally do not show a significant advantage in high‐dose regions of the target volume, but the 2ARC+OBSA+15 plan is closer to the clinical plan. However, in terms of OAR protection, RAD plans show clear advantages, especially for the bladder, rectum, spinal cord, and bilateral femoral heads. For the small intestine, the high‐dose region does not demonstrate an advantage in RAD plans. Only the 2ARC+OBSA+15, 2ARC+STAT+15, and RAD+QSG plans show similar dose sparing to the clinical plan.

### 3.2 Target dose statistics

**FIGURE 3:**
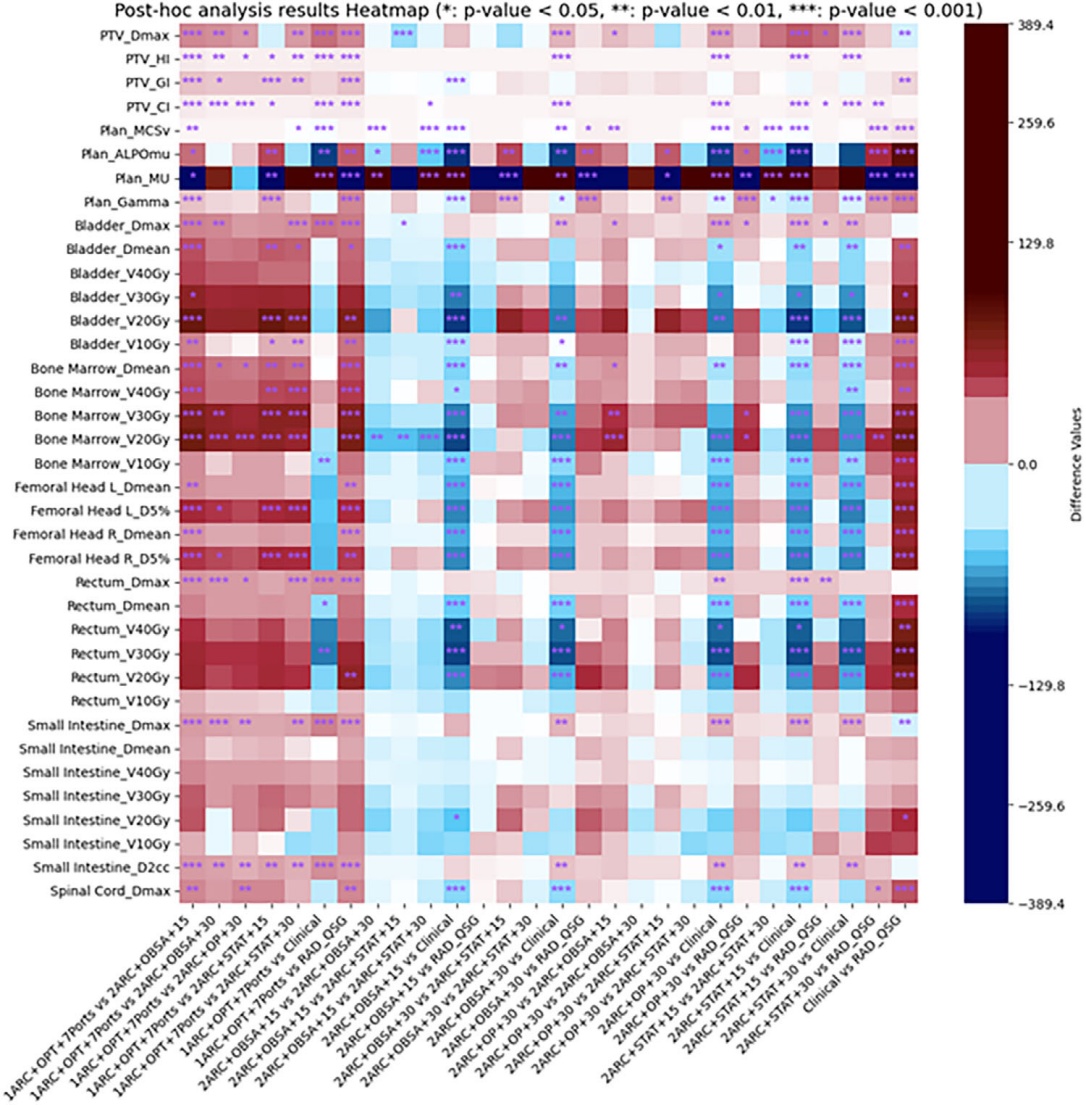
The post hoc analysis presents the results of all plans and dose metrics. The * indicates a *p*‐value <0.05, ** indicates a *p*‐value < 0.01, and *** indicates a *p*‐value < 0.001. The color represents the difference between two groups. For example, the color of group 1ARC+OPT+7Ports vs. 2ARC+OPT+30 represents (1ARC+OPT+7Ports) ‐ (2ARC + OPT + 30). A cold color indicates a value <0, while a hot color indicates a value >0.

For the statistical analysis of PTV dose metrics, specifically, when the datasets exhibited a normal distribution along with homogeneous variance, one‐way analysis of variance (ANOVA) and the post hoc LSD test were utilized. However, in cases where this was not the situation, the Kruskal–Wallis test and the Mann–Whitney *U* test were applied. The analyzed metrics encompassed $D_{max}$(Gy), CI, HI, and GI. For all RAD and clinical plans, the PTV prescription dose coverage was normalized to 95%. However, the RAD plans showed inferior dose homogeneity (HI) compared to the clinical plan. Additionally, the clinical plan exhibited a lowered $D_{max}$(Gy). Among the RAD plans, the 2ARC+OBSA+15 and RAD_QSG plans demonstrated significantly better dose homogeneity. Regarding dose fall‐off, although the 1ARC+OPT+7Ports plan performed the worst, the 2ARC + OBSA + 15 and RAD_QSG plans showed superior dose fall‐off compared to the clinical plan, offering a better dose gradient. Detailed results can be found in Table 2 and Figure 3.

**TABLE 2:** Comparative analysis of PTV dosimetric parameters across eight plans. Data presented as median (IQR).

Dose metrics	Dmax (Gy)	HI	GI	CI
1ARC+OPT+7Ports	51.51 (51.11, 51.82)	0.12 (0.11, 0.12)	3.86 (3.59, 3.94)	1.04 (1.03, 1.05)
2ARC+OPT+30	49.97 (49.47, 50.39)	0.08 (0.07, 0.09)	3.40 (3.27, 3.59)	1.00 (0.99, 1.00)
2ARC+OBSA+15	49.25 (49.10, 49.38)	0.07 (0.07, 0.08)	3.18 (3.07, 3.30)	0.99 (0.99, 1.00)
2ARC+OBSA+30	49.58 (49.45, 49.87)	0.08 (0.07, 0.08)	3.35 (3.27, 3.48)	1.00 (0.99, 1.00)
2ARC+STAT+15	52.47 (49.56, 54.85)	0.08 (0.07, 0.12)	3.26 (3.13, 3.44)	1.00 (0.99, 1.03)
2ARC+STAT+30	49.84 (49.46, 50.06)	0.08 (0.07, 0.09)	3.29 (3.20, 3.46)	1.01 (1.00, 1.01)
Clinical	48.58 (48.38, 48.78)	0.06 (0.06, 0.06)	3.56 (3.42, 3.66)	0.98 (0.98, 0.99)
RAD_QSG	49.47 (49.09, 49.86)	0.07 (0.07, 0.08)	3.24 (3.15, 3.30)	0.99 (0.99, 1.00)
*p*_value	<0.001	<0.001	<0.001	<0.001

Abbreviations: CI, Conformity Index; GI, Gradient Index; HI, Homogeneity Index; OBSA, optimize between static angles; OPT, optimize; STAT, static.

### 3.3 OAR dose statistics

While RAD plans do not provide a significant advantage in target dose homogeneity and the max dose, they excel in terms of OAR dose reduction. For the bladder, the mean dose ($D_{mean}$), $V_{30Gy}$(%) were significantly reduced in all RAD plans compared to the clinical plan, with reductions ranging from 8.29% to 13.05% and 26.98% to 32.46%, respectively. Similar advantages were observed for V_20_Gy and V_10_Gy across the RAD plans. Notably, the 2ARC+OBSA+15 and 2ARC+STAT+15 configurations demonstrated the most significant dose reductions of 26.48% and 26.71%, respectively, when compared to the clinical plan. For the rectum, RAD plans outperformed the clinical plan, with reductions in $D_{mean}$, $V_{20Gy}$ (%), $V_{30Gy}$ (%), and $V_{40Gy}$ (%) ranging from 9.21%–16.36%, 5.03%–17.51%, 18.24%–29.30%, and 23.73%–31.22%, respectively. The 2ARC+OBSA+15 and RAD_QSG plans provided additional advantages over other RAD plans. In terms of bone marrow dose sparing, except for the single‐arc RAD plan, which did not show significant benefits compared to the clinical plan, other RAD plans exhibited significant improvements, especially 2ARC+OBSA+15 and RAD_QSG. RAD plans generally showed no advantage in small intestine D2cc compared to the clinical plan, with the exception of the 2ARC+OBSA+15 and RAD_QSG plans which exhibited comparable doses. However, 2ARC+OBSA+15 and RAD_QSG Plans showed no significant difference compared to the clinical plan, suggesting that this plan has certain advantages in controlling the high dose to the small intestine in RAD plans. For other OARs such as bilateral femoral heads dose metrics and spinal cord $D_{max}$, RAD plans demonstrated significant dosimetric advantages over the clinical plan. The statistical values of some dosimetric parameters for OARs are presented in Table 3. For all the detailed statistical information about OARs, refer to Table S1. For a detailed statistical comparison of the differences, refer to Figure 3.

**TABLE 3:** Comparative analysis of OARs dosimetric parameters across eight planning groups. Data presented as mean ± SD or median (IQR) based on distribution.

Dose metrics	Bladder Dmean (Gy)	Bone marrow Dmean (Gy)	Femoral Head L D5% (Gy)	Femoral Head R D5% (Gy)	rectum Dmean (Gy)	Intestine D2cc (Gy)	Spinal Cord Dmax (Gy)
1ARC+OPT+7Ports	35.74 ± 3.27	27.76 (25.95, 29.26)	24.77 (22.29, 28.12)	22.83 (20.89, 28.25)	34.00 ± 3.02	48.50 (48.37, 49.15)	18.57 (17.90, 19.24)
2ARC+OPT+30	33.03 ± 2.72	25.21 (24.02, 26.04)	20.33 (18.11, 22.43)	18.80 (17.72, 22.09)	32.39 ± 3.26	47.37 (46.97, 47.48)	16.34 (16.12, 17.06)
2ARC+OBSA+15	31.47 ± 2.86	23.51 (22.90, 24.05)	17.85 (16.02, 19.84)	17.49 (15.42, 18.73)	31.62 ± 3.47	46.83 (46.72, 46.94)	16.32 (15.96, 17.04)
2ARC+OBSA+30	33.18 ± 3.19	25.05 (23.98, 25.65)	19.62 (17.41, 21.26)	18.38 (17.24, 19.41)	32.40 ± 3.33	47.17 (47.05, 47.38)	16.90 (16.02, 17.50)
2ARC+STAT+15	32.21 ± 4.33	24.42 (23.52, 25.23)	18.15 (15.59, 19.52)	16.50 (15.11, 19.02)	32.18 ± 3.69	47.16 (46.82, 48.66)	16.45 (15.98, 17.95)
2ARC+STAT+30	32.28 ± 3.09	24.79 (23.83, 25.59)	17.20 (16.04, 19.28)	16.90 (15.79, 18.09)	32.47 ± 3.31	47.41 (46.87, 47.60)	17.62 (16.85, 18.66)
Clinical	36.14 ± 2.87	27.81 (26.76, 28.96)	31.40 (27.53, 33.45)	30.62 (26.73, 33.28)	37.47 ± 2.29	46.53 (46.43, 46.79)	20.26 (18.94, 23.51)
RAD_QSG	32.49 ± 1.77	23.64 (23.34, 24.07)	18.54 (16.18, 19.75)	17.82 (16.50, 19.86)	31.34 ± 2.76	46.81 (46.75, 46.95)	16.32 (15.86, 16.89)
*p*‐value	<0.001	<0.001	<0.001	<0.001	<0.001	<0.001	<0.001

Abbreviations: OBSA, optimize between static angles; OPT, optimize; STAT, static.

### 3.4 Plan complexity and QA results

Multiple plan complexity measures were used to explore the differences between RAD and clinical plans. Compared to clinical plans, RAD plans exhibited significantly higher complexity, as evidenced by lower MCSv and ALPOmu values (*p* < 0.001). Despite having higher MU values (indicative of increased modulation), RAD plans maintained a gamma passing rate >97%, demonstrating comparable delivery accuracy to clinical plans. For example, the typical ARC+OBSA+15 and RAD_QSG plans have significant advantages over clinical and other RAD plans in terms of the dose sparing of OARs. However, they exhibit greater complexity in plan complexity and larger MU values. The detailed results are provided in Table 4 and Figure 3.

**TABLE 4:** Analysis of MU, plan complexity, and gamma verification passing rate for the eight planning groups. Data presented as median (IQR).

Plan metrics	MCSv	ALPOmu	MU	Gamma (%)
1ARC+OPT+7Ports	0.18 (0.18, 0.20)	21.08 (20.12, 24.68)	826.99 (773.10, 851.92)	99.26 (99.05, 99.34)
2ARC+OPT+30	0.19 (0.18, 0.20)	20.56 (19.70, 21.72)	833.31 (815.42, 845.35)	99.00 (98.89, 99.17)
2ARC+OBSA+15	0.17 (0.16, 0.17)	18.16 (17.55, 19.43)	884.70 (864.33, 903.98)	98.65 (98.47, 98.75)
2ARC+OBSA+30	0.19 (0.18, 0.20)	21.40 (20.06, 23.36)	810.09 (781.57, 834.38)	98.98 (98.92, 99.19)
2ARC+STAT+15	0.18 (0.14, 0.19)	16.75 (14.82, 18.72)	938.99 (880.40, 1207.15)	98.08 (97.49, 98.39)
2ARC+STAT+30	0.23 (0.22, 0.25)	24.20 (21.54, 26.06)	750.08 (725.09, 787.17)	98.90 (98.71, 99.12)
Clinical	0.27 (0.25, 0.28)	44.67 (41.36, 48.05)	549.55 (517.67, 576.15)	99.66 (99.49, 99.82)
RAD_QSG	0.17 (0.17, 0.18)	17.52 (16.64, 18.04)	926.90 (903.06, 955.80)	97.22 (97.02, 97.44)
*p*‐value	<0.001	<0.001	<0.001	<0.001

Abbreviations: ALPOmu, average leaf pair opening with MU. For the MCSv and ALPOmu, the lower values mean the higher plan complexity; MCSv, modulation complexity score of VMAT; MU, monitor unit; OBSA, optimize between static angles; OPT, optimize; STAT, static.

## 4 DISCUSSION

The hybrid planning approach has shown capability in reducing OAR doses, as evidenced by Zhao N et al. and Ugur Akbas et al. in their studies on nasopharyngeal cancer. Their findings demonstrated dosimetric advantages in hybrid planning compared to VMAT or IMRT, with reductions in OAR doses such as TMJ by up to 12.8%.^6^, ^7^ In cervical cancer, Martín‐Tovar EA et al. observed that hybrid plans could reduce $V_{50Gy}$ for the rectum, bladder, and bowel by 54%, 25.5%, and 38.8%, respectively, compared to VMAT.^9^ Additionally, collimator angle optimization in VMAT has been shown to effectively limit high dose outside the target volume and reduce the OAR dose.^14^, ^15^ The RAD technique introduces significant innovations to VMAT by allowing gantry pauses at some specific beam directions to deliver additional dose and MLC modulations, called static angle modulated ports (STAMPs). Unlike traditional hybrid plans that combine VMAT and IMRT techniques, RAD achieves this by incorporating fixed beam angles with additional control points. Moreover, RAD facilitates collimator rotation during beam delivery, enhancing MLC utilization and increasing RT plan modulation capabilities.

RAD technology exhibits two particularly salient features. Firstly, it directly and manually applies STAMPs to the VMAT radiation fields, akin to a hybrid plan. The second feature is the dynamic rotation of the collimator within the VMAT radiation fields. Especially for the dynamic collimator rotation, it supports three modes. One mode is to optimize the collimator angle completely based on the shape of the target. Another mode is to manually set the starting and ending points of the collimator rotation according to experience, and specific collimator angles can be anchored at different STAMPs. Thus, the optimization of the collimator occurs between the anchored angles along with the dynamic rotation of the gantry. The last mode is like conventional VMAT technology, where a specific static collimator angle is used to optimize the VMAT plan. The optimization of the collimator angle adheres to the principle of maximizing the utilization efficiency of MLC leaves. Specifically, this involves minimizing the modulation movement range of each pair of leaves as well as the rotation angle of the collimator.

This research shows that the main apertures were 15 and 30 cm. There are some reasons. The maximum extension of a single‐side leaf of the Varian Millennium MLC or HDMLC from the carriage is merely 15 cm. During plan execution, the MLC carriage remains stationary. To guarantee maximum modulation capacity of both MLC sides during plan optimization, it's essential to control the left‐right aperture size at no more than 15 cm. In fact, for RAD and conventional VMAT plans, setting the aperture to 15 cm allows the bilateral MLC to reach its peak in‐field modulation capacity. So, in clinical practice, when the target size in the radiation‐field projection direction exceeds 15 cm, a two‐arc approach is used. An appropriate collimator angle is assigned to ensure the target is within the two radiation fields in any direction. In clinical practice, conventional VMAT may employ apertures larger than 15 cm. However, for RAD planning, constraining the aperture to 15 cm is a critical parameter. This setting capitalizes on the dynamic collimator rotation to maximize the modulation efficiency of both MLC leaf banks, which is a fundamental dosimetric advantage of the technique. Considering the MLC's characteristic two‐sided modulation capabilities, a maximum 30 cm aperture setting is introduced as a planning approach. The purpose of the 1 arc RAD plan is to explore whether a single arc RAD irradiation method can achieve the same effect as a conventional two arc clinical plan. The settings of STAMPs mainly refer to those of IMRT radiation fields. However, since the left‐right width of the cervical cancer target area is greater than 15 cm, the method of automatically setting the aperture is adopted >15 cm. The application of the single arc RAD plan based on this setting in cervical cancer is a preliminary attempt. More settings related to the two‐arc RAD plan will be carried out in the future. Different collimator motion types (OPT, OBSA, STAT) are governed by specific optimization principles, primarily focusing on maximizing MLC efficiency. Therefore, selecting the appropriate collimator mode is a critical consideration in the planning process.

For PTV dosimetric parameters, RAD plans ensured 95% coverage for all targets but exhibited disadvantages in HI and CI. While CI differences were minimal (except for the 1ARC+7F plan), the addition of static fields in the 1ARC+7F plan slightly affected target conformity. Other RAD plans, mainly 2ARC‐based, added two fixed fields with almost the same orientations, resulting in less impact on CI. However, due to the relaxed constraints on high‐dose regions during optimization, RAD plans demonstrated slightly lower uniformity compared to clinical plans. In terms of dose fall‐off, RAD plans with a 15 cm aperture and optimized collimator angles showed distinct advantages. By optimizing collimator angles and setting an optimal aperture size, RAD improved MLC efficiency, reduced irradiation to tissues outside the target, and achieved steeper dose gradients. Similar findings have been reported by Lee CM et al. and G.G.Y. Kim et al., who highlighted the ability of dynamic collimator angle to reduce high‐dose regions surrounding the target.^14^, ^25^

RAD plans demonstrated distinctive advantages for target volumes with geometrically complex, concave shapes that are in close proximity to multiple OARs. For parallel structures such as the bladder and rectum, RAD plans achieved significant dose reductions, with $V_{30Gy}$ decreased by up to 32.46% and 29.03%, respectively. Because the optimization of the collimator is based on the direction of the target volume, it is possible to reduce the moving distance of the MLC during the VMAT beam delivery and maximize the efficiency of the MLC. STAMPs technology also contributed to avoiding OARs, further reducing their dose exposure. Among the seven RAD optimization methods, the 2ARC+STAT+15, RAD_QSG and 2ARC+OBSA+15 plans stood out, with 2ARC+OBSA+15 showing superior performance in most cases. In addition, a type of STAMPs based on specific angles and quantities (RAD_QSG) also demonstrates the same advantage in OAR dose sparing as the ARC+OBSA+15 plan. There is no significant statistical difference, but in some numerical values, the ARC+OBSA+15 plan has more advantages. In summary, the 15 cm aperture provided a balance between maximizing MLC modulation and maintaining mechanical constraints, enhancing overall plan efficiency.

For MU and plan complexity, RAD plans were less advantageous compared to clinical plans. However, given modern linear accelerators' high beam and mechanical precision, the impact of increased MU and complexity was less critical. Despite higher MU values, RAD plans maintained high gamma pass rate (>97%), underscoring their clinical feasibility. The increase in MU and complexity can be attributed to improved MLC modulation efficiency and the addition of static gantry angle ports. Nevertheless, the dosimetric benefits for OARs outweighed these disadvantages. This study has limitations in plan validation. While secondary dose verification provided higher dose accuracy validation, it could not account for MLC positional accuracy. Additionally, it could not account for the synchrony between MLC motion and collimator rotation, nor for output dose variations from the linear accelerator.^26^

In this research, high‐dose regions within the target were not strictly constrained, leading to slightly reduced PTV homogeneity. However, the superior dose constraints for OARs demonstrated the advanced capabilities and performance improvements of RAD planning methods. RAD plans include multiple adjustable variables, such as number of arcs, collimator rotation range, number and placement of STAMPs aperture size setting, and optimization STAMPs weighting. This study employed six preliminary optimization methods, which, while not comprehensive, revealed that controlling the collimator angle optimization range and setting the aperture to 15 cm tended to yield optimal results. In the process of optimizing RAD plans, as opposed to conventional VMAT plans, additional variables such as STAMPs, collimator rotation, and aperture size are introduced. These variables can be configured in a multitude of ways, giving rise to a profusion of combinations in clinical scenarios. This study centers on the outcomes associated with the settings of aperture size, collimator rotation, and specific angles of STAMPs. The primary experience of this study is derived from VMAT plan‐design endeavors. Consequently, most of the plans utilize a double‐arc configuration, and the STAMPS are predominantly set at the patient's posterior. This method can, to the greatest extent possible, ensure a more regular dose distribution shape. Additionally, it can enhance the modulation capacity within the rectal region, aiming for improved protection of the rectum. However, it is infeasible to comprehensively encompass all parameter settings of RAD plans. Consequently, there remain numerous aspects in clinical practice that both can and ought to be explored continuously.

Our study prioritized collimator angle and field size optimization, consequently deferring a comprehensive exploration of port quantity impact. Future work should establish the optimal balance between port count, dosimetric performance, and deliverability for these techniques. Additionally, considering the features of the RAD plan, a high‐quality plan should be achievable with 1ARC combined with some STAMPs, along with an optimal aperture size setup and collimator rotation option. In this research, setting only one 1ARC option might not be sufficient; the 1 arc RAD plan also warrants further exploration.

## 5 CONCLUSION

The preliminary exploration of RAD plans demonstrates a notable advantage in OARs dose sparing, despite an increase in plan complexity. Notably, configurations with 15 cm jaw tracking range and collimator angle optimizations within specific intervals yielded superior results. Therefore, it is highly significant for studies to further explore different mechanical axis combinations in the future, as well as the 1 arc RAD plan method.

## AUTHOR CONTRIBUTIONS

**Xiangyin Meng**: Investigation (equal); data curation (lead); formal analysis (equal); validation (lead); writing—original draft (lead). **Zhiqun Wang**: Methodology and validation (equal). **Yiming Zhang**: Methodology (equal); investigation (equal). **Xingliu Wang**: Conceptualization (lead); formal analysis (lead); writing—original draft (equal). **Xiaoshen Wang**: Formal analysis (equal); visualization (lead). **Weihua Zhu**: Formal analysis (equal); visualization (equal). **Fuqiang Chen**: Formal analysis (supporting); visualization (supporting). **Zhen Zhang**: resources (equal). **Fengchao Xu**: Software (lead); conceptualization (supporting). **Zhufeng Wu**: Software (equal); resources (equal). **Bo Yang**: conceptualization (equal); supervision (lead); project administration (lead). **Jie Qiu**: Funding acquisition (lead).

## CONFLICT OF INTEREST STATEMENT

The authors declare no conflicts of interest.

## Supporting information



Supporting information

## Data Availability

Research data are available and can be obtained by contacting the corresponding author.
