# Supplementary material for: RapidArc Dynamic (RAD) multi‐mechanical axis optimization achieves enhanced OAR sparing in cervical cancer: A dosimetric comparison study
Source: J Appl Clin Med Phys. 2026 Feb 17;27(2):e70506. doi: 10.1002/acm2.70506 (PMC12912941; doi:10.1002/acm2.70506)
Supplement: Supplementary file 1 — Supporting information [file ACM2-27-e70506-s001.docx]

Supplementary Table 1. Comparative analysis of Target or OARs dosimetric parameters across eight planning groups. Data presented as mean ± SD or median (IQR) based on distribution.

| **Target/OARs** | **Dose Metrics** | **1ARC+OPT+7Ports** | **2ARC+OPT+30** | **2ARC+OBSA+15** | **2ARC+OBSA+30** | **2ARC+STAT+15** | **2ARC+STAT+30** | **Clinical** | **RAD_QSG** | **P_Value** |
| --- | --- | --- | --- | --- | --- | --- | --- | --- | --- | --- |
| PTV | Dmax(Gy) | 51.51(51.11,51.82) | 49.97(49.47,50.39) | 49.25(49.10,49.38) | 49.58(49.45,49.87) | 52.47(49.56,54.85) | 49.84(49.46,50.06) | 48.58(48.38,48.78) | 49.47(49.09,49.86) | <0.001 |
|  | HI | 0.12(0.11,0.12) | 0.08(0.07,0.09) | 0.07(0.07,0.08) | 0.08(0.07,0.08) | 0.08(0.07,0.12) | 0.08(0.07,0.09) | 0.06(0.06,0.06) | 0.07(0.07,0.08) | <0.001 |
|  | GI | 3.86(3.59,3.94) | 3.40(3.27,3.59) | 3.18(3.07,3.30) | 3.35(3.27,3.48) | 3.26(3.13,3.44) | 3.29(3.20,3.46) | 3.56(3.42,3.66) | 3.24(3.15,3.30) | <0.001 |
|  | CI | 1.04(1.03,1.05) | 1.00(0.99,1.00) | 0.99(0.99,1.00) | 1.00(0.99,1.00) | 1.00(0.99,1.03) | 1.01(1.00,1.01) | 0.98(0.98,0.99) | 0.99(0.99,1.00) | <0.001 |
| Bladder | Dmax(Gy) | 50.69(50.23,51.57) | 49.12(48.52,49.48) | 48.28(48.16,48.43) | 48.67(48.46,48.83) | 48.89(48.48,49.96) | 48.78(48.24,49.11) | 47.94(47.87,48.12) | 48.20(48.06,48.55) | <0.001 |
|  | Dmean(Gy) | 35.74±3.27 | 33.03±2.72 | 31.47±2.86 | 33.18±3.19 | 32.21±4.33 | 32.28±3.09 | 36.14±2.87 | 32.49±1.77 | <0.001 |
|  | V40Gy(%) | 44.36±9.66 | 40.68±8.00 | 39.64±7.99 | 40.78±8.32 | 41.45±10.27 | 41.24±8.35 | 44.98±9.55 | 41.02±7.14 | <0.487 |
|  | V30Gy(%) | 66.98±12.91 | 58.04±9.92 | 55.21±9.90 | 59.15±11.68 | 57.45±14.28 | 58.20±10.42 | 70.05±13.37 | 58.13±6.74 | <0.001 |
|  | V20Gy(%) | 90.66(85.16,96.13) | 79.54(75.70,85.06) | 69.50(65.96,78.64) | 79.98(73.40,87.28) | 69.27(65.61,76.93) | 74.69(69.26,79.31) | 94.42(92.10,98.38) | 75.57(72.86,80.14) | <0.001 |
|  | V10Gy(%) | 100.00(99.99,100.00) | 100.00(99.22,100.00) | 97.64(94.95,99.50) | 99.80(97.07,100.00) | 98.92(92.16,99.98) | 98.86(95.14,99.74) | 100.00(100.00,100.00) | 97.35(96.27,99.44) | <0.001 |
| Bone Marrow | Dmean(Gy) | 27.76(25.95,29.26) | 25.21(24.02,26.04) | 23.51(22.90,24.05) | 25.05(23.98,25.65) | 24.42(23.52,25.23) | 24.79(23.83,25.59) | 27.81(26.76,28.96) | 23.64(23.34,24.07) | <0.001 |
|  | V40Gy(%) | 17.59(14.46,20.97) | 14.75(12.46,16.24) | 13.22(11.59,14.51) | 14.51(12.40,15.90) | 13.28(11.76,15.09) | 12.87(11.23,14.13) | 16.23(14.52,18.70) | 12.64(11.89,13.78) | <0.001 |
|  | V30Gy(%) | 43.42(36.04,49.74) | 34.45(30.32,37.57) | 28.73(26.58,30.46) | 32.49(30.26,35.03) | 30.61(28.75,34.21) | 30.68(28.18,33.06) | 42.69(38.06,46.49) | 29.53(27.15,30.96) | <0.001 |
|  | V20Gy(%) | 72.92±7.51 | 62.31±6.46 | 53.63±5.45 | 61.28±5.90 | 60.96±7.14 | 63.51±6.15 | 76.06±5.04 | 56.36±4.64 | <0.001 |
|  | V10Gy(%) | 91.49±3.11 | 90.08±2.49 | 89.63±3.41 | 91.13±2.53 | 90.15±3.42 | 91.36±3.64 | 95.20±1.68 | 88.82±2.85 | <0.001 |
| Femoral Head L | Dmean(Gy) | 11.74(10.72,12.37) | 10.43(9.77,11.13) | 9.88(9.54,10.40) | 10.38(9.83,10.76) | 10.41(9.88,10.86) | 10.72(10.29,11.30) | 18.94(15.96,21.53) | 9.87(9.41,10.72) | <0.001 |
|  | D5%(Gy) | 24.77(22.29,28.12) | 20.33(18.11,22.43) | 17.85(16.02,19.84) | 19.62(17.41,21.26) | 18.15(15.59,19.52) | 17.20(16.04,19.28) | 31.40(27.53,33.45) | 18.54(16.18,19.75) | <0.001 |
| Femoral Head R | Dmean(Gy) | 11.62(10.67,12.33) | 10.40(9.71,10.61) | 9.83(9.56,10.23) | 10.39(9.76,10.67) | 10.18(9.85,10.78) | 10.22(9.98,10.61) | 18.93(16.69,20.86) | 9.85(9.53,10.24) | <0.001 |
|  | D5%(Gy) | 22.83(20.89,28.25) | 18.80(17.72,22.09) | 17.49(15.42,18.73) | 18.38(17.24,19.41) | 16.50(15.11,19.02) | 16.90(15.79,18.09) | 30.62(26.73,33.28) | 17.82(16.50,19.86) | <0.001 |
| Rectum | Dmax(Gy) | 49.66(48.85,50.07) | 48.17(47.94,48.50) | 47.88(47.71,48.10) | 47.97(47.74,48.40) | 48.41(47.97,49.78) | 48.05(47.87,48.39) | 47.72(47.52,47.88) | 47.76(47.64,48.08) | <0.001 |
|  | Dmean(Gy) | 34.00±3.02 | 32.39±3.26 | 31.62±3.47 | 32.40±3.33 | 32.18±3.69 | 32.47±3.31 | 37.47±2.29 | 31.34±2.76 | <0.001 |
|  | V40Gy(%) | 44.01(36.90,52.65) | 40.70(34.49,50.61) | 38.35(34.03,50.62) | 40.87(34.82,50.43) | 39.30(34.31,50.70) | 41.71(36.89,52.12) | 57.86(49.11,68.11) | 40.80(33.97,49.45) | 0.003 |
|  | V30Gy(%) | 66.42±11.75 | 60.65±12.28 | 58.38±12.48 | 60.83±12.16 | 59.83±12.67 | 62.26±11.94 | 81.39±7.51 | 57.54±10.44 | <0.001 |
|  | V20Gy(%) | 85.36(78.80,87.86) | 80.08(72.49,83.55) | 76.57(68.54,81.90) | 80.78(74.26,83.82) | 78.18(72.14,83.88) | 79.85(73.74,81.27) | 89.84(87.74,92.98) | 74.11(70.49,77.50) | <0.001 |
|  | V10Gy(%) | 91.27±3.98 | 90.83±3.84 | 90.15±3.94 | 90.80±4.22 | 90.92±4.26 | 91.76±4.14 | 93.12±4.06 | 90.34±3.49 | <0.362 |
| Small Intestine | Dmax(Gy) | 50.02(49.63,50.55) | 48.76(48.41,49.13) | 48.49(48.18,48.67) | 48.62(48.46,48.86) | 48.78(48.12,50.53) | 48.69(48.14,49.26) | 47.63(47.36,47.84) | 48.59(48.27,48.89) | <0.001 |
|  | Dmean(Gy) | 19.76±2.64 | 18.95±2.21 | 18.44±2.02 | 19.37±2.09 | 18.89±2.08 | 19.65±2.20 | 19.83±2.56 | 18.55±1.76 | <0.295 |
|  | V40Gy | 18.22±5.96 | 16.72±5.30 | 16.12±5.23 | 16.61±5.22 | 16.78±5.20 | 16.93±5.18 | 17.11±5.80 | 16.48±5.31 | <0.967 |
|  | V30Gy | 31.21±7.51 | 29.04±6.71 | 27.46±6.30 | 29.75±6.14 | 27.97±6.23 | 28.88±6.45 | 29.56±8.16 | 27.90±6.19 | <0.711 |
|  | V20Gy | 43.50±7.46 | 41.47±6.39 | 39.59±5.01 | 43.90±6.43 | 40.68±5.90 | 43.37±6.70 | 46.42±8.05 | 40.18±4.42 | 0.015 |
|  | V10Gy | 60.24±7.09 | 59.54±7.45 | 59.09±7.58 | 60.61±6.72 | 60.25±6.79 | 63.45±6.73 | 62.87±6.40 | 58.42±6.01 | <0.247 |
|  | D2cc(Gy) | 48.50(48.37,49.15) | 47.37(46.97,47.48) | 46.83(46.72,46.94) | 47.17(47.05,47.38) | 47.16(46.82,48.66) | 47.41(46.87,47.60) | 46.53(46.43,46.79) | 46.81(46.75,46.95) | <0.001 |
| Spinal Cord | Dmax(Gy) | 18.57(17.90,19.24) | 16.34(16.12,17.06) | 16.32(15.96,17.04) | 16.90(16.02,17.50) | 16.45(15.98,17.95) | 17.62(16.85,18.66) | 20.26(18.94,23.51) | 16.32(15.86,16.89) | <0.001 |
